# Trustworthy artificial intelligence and the European Union AI act: On the conflation of trustworthiness and acceptability of risk

**DOI:** 10.1111/rego.12512

**Published:** 2023-02-06

**Authors:** Johann Laux, Sandra Wachter, Brent Mittelstadt

**Affiliations:** ^1^ Oxford Internet Institute University of Oxford 1 St Giles Oxford OX1 3JS UK

**Keywords:** AI act, artificial intelligence, regulation, risk, trust

## Abstract

In its AI Act, the European Union chose to understand trustworthiness of AI in terms of the acceptability of its risks. Based on a narrative systematic literature review on institutional trust and AI in the public sector, this article argues that the EU adopted a simplistic conceptualization of trust and is overselling its regulatory ambition. The paper begins by reconstructing the conflation of “trustworthiness” with “acceptability” in the AI Act. It continues by developing a prescriptive set of variables for reviewing trust research in the context of AI. The paper then uses those variables for a narrative review of prior research on trust and trustworthiness in AI in the public sector. Finally, it relates the findings of the review to the EU's AI policy. Its prospects to successfully engineer citizen's trust are uncertain. There remains a threat of misalignment between levels of actual trust and the trustworthiness of applied AI.

## 1 Introduction

The global race to establish technological leadership in artificial intelligence (AI) is escorted by an effort to develop “trustworthy AI.” Numerous policy frameworks and regulatory proposals make principled suggestions as to which features render AI “trustworthy” [Cf. the overviews in Lucia Vesnic‐Alujevic et al., 2020 and Thiebes et al., 2021], Private companies such as auditing firms are offering their clients support in designing and deploying “trustworthy AI” (Mökander & Floridi, 2021). The emphasis on trustworthiness serves a strategic purpose: induce people to place trust in AI so that they will use it more and, hence, unlock the technology's economic and social potential.

This strategy is not unfounded. Trust cannot be created on command. Signaling trustworthiness is thus the most promising option for regulators and technologists who seek to create the initial trust needed for a broader uptake of AI (Drake et al., 2021; O'Neill, 2012). Success, however, is not guaranteed. Even allegedly trustworthy persons, institutions, and technologies might not be trusted after all. For example, populations which have historically faced discrimination may reasonably distrust broadly accepted signals of trustworthiness (Scheman, 2020).

As part of the global trustworthiness effort, the European Commission recently proposed a legal framework for trustworthy AI, the “AI Act” (European Commission, 2021b). The AI Act explicitly pursues the dual purpose of promoting the uptake of the technology and addressing the risks associated with its use (AI Act, Recital 81 and p. 1). At the time of writing, the proposal is being discussed by the Council of the European Union and the European Parliament, both of which must agree on a common text before the AI Act can pass into law.^1^

As this article will show, in its proposal the Commission chose to understand “trustworthiness” narrowly in terms of the “acceptability” of AI's risks, with the latter being primarily assessed through conformity assessments carried out by technology experts (see Section 2.1). This regulatory conflation of trustworthiness with the acceptability of risks invites further reflection.

Based on a systematic narrative literature review on trust research, this article argues that the European Union (EU) is overselling its regulatory ambition and oversimplifying a highly complex and heterogeneous set of closely related concepts. First, while there is an inherent relationship between trust, trustworthiness, and the perceived acceptability of risks (Poortinga & Pidgeon, 2005), the AI Act will itself require citizens' trust to succeed in promoting the uptake of AI. Second, the concept of trustworthiness serves an important normative function. It allows to assess whether people's actual levels of trust are normatively “justified” (Cf. Lee, 2022) or “well‐placed.” This justification depends on whether their degree of trust in something matches its degree of trustworthiness. A person's trust can be “blind” or misplaced; so too can their mistrust. There is a rich philosophical debate as to whether AI even has the capacity of being a genuine object of trust. Its lack of human qualities such as intentionality could prohibit such attributions. AI may then be merely reliable, but not trustable [Miller & Freiman, 2020; for the debate, see further Rieder et al. (2021), Weydner‐Volkmann and Feiten (2021), Ryan (2020), Grodzinsky et al. (2020), Nickel et al. (2010), and Taddeo (2009)].

Conflating trust and trustworthiness with the acceptability of risks blurs the distinction between acceptability judgments made by domain experts and the trustworthiness of AI systems implemented in society. Others have criticized before that the AI Act outsources decisions about which risks are “acceptable” to AI providers with an economic interest to market the AI system (Smuha et al., 2021). Rather than providing a seal of approval, we argue that trustworthiness is a longitudinal concept that necessitates an iterative process of controls, communication, and accountability to establish and maintain its existence across both AI technologies and the institutions using them. The AI Act suggests an unfounded bright‐line distinction between acceptable and unacceptable risks and hence trustworthy and non‐trustworthy AI. This approach is incompatible with the conceptualization of trustworthiness as a longitudinal process as opposed to a binary characteristic of systems and the risks they pose. This article therefore aims to provide an intervention into the EU's policy effort to develop “trustworthy AI” by risk regulation based on a review of the multi‐disciplinary literature on trust. Instead of working out a coherent theory of trust, it aims to demonstrate the conceptual futility of labeling a complex AI system “trustworthy” prior to placing it on the market.

We limit our analysis to the use of AI in public institutions. The potential of AI for the public sector is rapidly gaining interest (Gesk & Leyer, 2022; see also de Sousa et al., 2019). AI systems have already been introduced in public institutions (Desouza et al., 2017), with promises of higher quality services and increased efficiency (Sun & Medaglia, 2019). At the same time, AI's characteristics have led to considerable debate about whether and how the public sector should deploy the technology (Green, 2022). Many AI systems “reason by association”: they detect statistical patterns in data but do not offer causal explanations (Bishop, 2021). In addition, an AI system might include so many parameters that its outcome is opaque, resembling a “black box.” There is too much information to interpret its outcome clearly (Dignum, 2019). These features arguably set AI systems aside from other digital technologies already in use by public institutions.^2^

Through the proposed AI Act and other instruments, the European Commission nevertheless seeks to “make the public sector a trailblazer for using AI” (European Commission, 2021a). Its 2020 “White Paper” on AI (European Commission, 2020) holds it “essential” that the public sector, especially in healthcare and transport, begins to “rapidly” deploy products and services that rely on AI (White Paper, p. 8). The European Commission also supports the uptake of AI in the domain of justice (European Commission, 2018).

While making AI trustworthy has garnered substantial political momentum, equal attention needs to be paid to AI's potential to erode the trustworthiness of public institutions and, with it, their own ability to produce trust in the population (Bodó, 2021). Without trust, the public sector risks losing support and compliance by citizens.

Some publicly documented uses of automated decision‐systems have led to widespread criticism and the cessation of operations. Consider, for example, the algorithmic prediction of social welfare fraud in marginalized neighborhoods in the Netherlands or the algorithmic profiling of families for early detection of vulnerable children in Denmark (Kayser‐Bril, 2020; Vervloesem, 2020). AI in the public sector can quickly become politicized, not least because of the public sector's dual role. It is at the same time drawn to using AI to increase its efficiency and under an obligation to protect citizens from harm caused by AI (Kuziemski & Misuraca, 2020).

Citizens' concerns about AI in the public sector have likewise been identified as one of the major obstacles to broader implementation (Gesk & Leyer, 2022, pp. 1–2). However, while the use of (non‐AI‐based) information and communication technology in the public sector has been widely researched—often under the rubric of “eGovernment”—the use of AI in the public sector and its acceptance by citizens is still understudied [Gesk & Leyer, 2022; drawing on Sun and Medaglia (2019); Wang and Liao (2008)]. At the same time, insights gained from the private sector cannot easily be transferred to the public sector, not least because the latter's target is not to maximize profits generated from customers [See the references in Gesk and Leyer (2022, p. 1)]. Moreover, public services' adoption of AI further differs from the private sector as it can have a coercive element. Citizens will often have no choice but to use and pay for the services (through taxes or insurance premiums) whether or not they prefer an AI system to be involved (Aoki, 2021). As the coercive power of public authority requires justification (Simmons, 1999), AI in the public sector thus also raises questions of legitimacy.

Politicization can add further justificatory pressure. Trust researchers consider how in highly politicized contexts of AI implementation, conflicts about what constitutes a “right” or “fair” decision are likely to erupt (de Bruijn et al., 2022; drawing on Bannister & Connolly, 2011b). The stakes of implementing AI in public services are thus high, invoking the foundational concepts of trust in and legitimacy of public authority.

This article proceeds as follows. Section 2 begins with a trust‐theoretical reconstruction of the conflation of “trustworthiness” with the “acceptability of risks” in the EU's AI policy. We then turn to our review of the literature on trust in AI implemented within public institutions. One simple definition of “trust” is the willingness of one party to expose themselves to a position of vulnerability towards a second party under conditions of risk and uncertainty as regards the intentions of that second party (similarly, Bannister & Connolly, 2011b, p. 139). However, the term “trust” has found multiple definitions within and across social science disciplines, so much that the state of defining trust has been labeled as one of “conceptual confusion” (McKnight & Chervany, 2001). This makes comparing and evaluating trust research across disciplines (and sometimes even within one discipline) extremely difficult.

Section 3, therefore, develops a prescriptive set of variables for reviewing trust‐research in the context of AI. We differentiate between normative and empirical research as well as between subject, objects, and roles of trust. Section 4 then uses those variables as a structure for a narrative review of prior research on trust and trustworthiness in AI in the public sector. We identify common themes in the reviewed literature and reflect on the heterogeneity of the field and, thus, the many ways in which trust in AI can be defined, measured, incentivized, and governed.

This article concludes in Sections 5 and 6 by relating the findings of the literature review to the EU's AI policy and especially its proposed AI Act. It states the uncertain prospects for the AI Act to be successful in engineering citizens' trust. There remains a threat of misalignment between levels of actual trust and the trustworthiness of applied AI. The conflation of “trustworthiness” with the “acceptability of risks” in the AI Act will thus be shown to be inadequate.

## 2 A reconstruction of trustworthiness as acceptability of risks in the AI act

The effort to develop “trustworthy AI” through regulatory laws such as the AI Act acknowledges a need for AI to be trusted if it is to be widely adopted. This obviously presupposes AI to be a possible object of trust. As mentioned, some philosophers deny this possibility. In policy, however, trust is considered a key element in relationships between humans as well as humans and technology (Robinson, 2020). To capture this regulatory intention, we thus pursue an inclusively broad scope for trust relationships in this article, including human trust in AI. The High‐Level Expert Group on Artificial Intelligence's (AI HLEG), an expert group appointed to advise the European Commission on its AI strategy,^3^ identifies “trust” in its 2019 Ethics Guidelines for Trustworthy AI (High‐Level Expert Group on Artificial Intelligence, 2019) (herein: “Guidelines”) as nothing less than the “bedrock of societies” (Guidelines, p. 4). Research finds trust to be associated with positive developments such as higher levels of economic growth, higher levels of civic engagement, higher quality government, and lower levels of crime and corruption [See the references in van Ingen and Bekkers (2015); Bjørnskov (2017)].

Policymakers thus have strong incentives to try and engineer trust. This section reconstructs this attempt visible in the AI Act. It begins by showing that the European Commission explicitly chose to understand “trustworthiness” in terms of the acceptability of risks (Section 2.1). We then draw on risk research to highlight how trust and the perceived acceptability of risks have been found to be highly correlated in empirical studies (Section 2.2). We then shift to a normative perspective and differentiate procedural from substantive elements of trustworthiness (Section 2.3). We conclude this section by formulating two research questions for this article (Section 2.4).

### 2.1 The EU AI act: Trustworthiness as acceptability of risks

In its 2020 White Paper, the European Commission explicitly states that trustworthiness is a “prerequisite” for AI's uptake in Europe (White Paper, p. 1 and p. 3). Lack of trust is identified as “a main factor holding back a broader uptake of AI” (White Paper, p. 9). The White Paper envisions a future regulatory framework for AI which is supposed to create an “ecosystem of trust” which in turn “should give citizens the confidence to take up AI applications” (White Paper, p. 3).

The Commission's 2021 AI Act proposal aims at implementing the White Paper's trust ecosystem by delivering a regulatory framework for the development of trustworthy AI (AI Act, p. 1). The AI Act adopts the White Paper's “twin objective” of promoting the uptake of the technology and of addressing the risks associated with using AI (AI Act, p. 1 and Recital 81). To ensure “a high level of trustworthiness of high‐risk AI systems, those systems should be subject to a conformity assessment prior to their placing on the market or putting into service” (AI Act, Recital 62). These conformity assessments follow the AI Act's “risk‐based approach” (AI Act, p. 13), which relies heavily on the notion of acceptability: AI applications which create “unacceptable” risks are prohibited (AI Act, p. 12 and Recital 27). Title II of the AI Act comprises a list of prohibited AI practices which are “considered *unacceptable* as contravening Union values, for instance by violating fundamental rights” (AI Act, p. 12, emphasis added). Residual risks in high‐risk AI systems must be “*acceptable*” (Art. 9(4) AI Act, emphasis added). “Certain mandatory requirements” are supposed to ensure that high‐risk AI systems “do not pose *unacceptable* risks to important Union public interests as recognized and protected by Union law” (AI Act, Recital 27, emphasis added).

Interestingly, the notion of the acceptability of risks was still absent in the Commission's White Paper. It can be found, however, playing a central role in the AI HLEG's Guidelines which define “trustworthy AI” as being lawful, ethical, and (technically as well as socially) robust (Guidelines, p. 2). They postulate seven key requirements for “trustworthy” AI: human agency and oversight; technical robustness and safety; privacy and data governance; transparency; diversity, non‐discrimination, and fairness; societal and environmental well‐being; accountability (Guidelines, p. 14).

Acceptability appears to be a key element for operationalizing these requirements. For example, as regards technical robustness the Guidelines state that “AI systems be developed […] in a manner such that they reliably behave as intended while minimizing unintentional and unexpected harm, and preventing *unacceptable* harm” (Guidelines, p. 16, emphasis added). If ethical principles must be balanced against each other in the context of AI, the Guidelines acknowledge that in some situations “no ethically *acceptable* trade‐offs” will be identifiable, as certain fundamental rights such as human dignity are “absolute and cannot be subject to a balancing exercise” (Guidelines, p. 13, emphasis added). In such cases, the “development, deployment, and use of the AI system should not proceed in that form” (Guidelines, p. 20). In turn, this suggests that acceptability is otherwise the threshold also for ethical AI. The overall importance of the acceptability threshold for “trustworthy AI” is reflected in the Guideline's Trustworthy AI Assessment List: AI developers should consider whether they estimate the likely impact of a failure of their AI when it “provides societally *unacceptable* results (for example discrimination)” and whether they command ways to measure if their system is “making an *unacceptable* amount of inaccurate predictions” (Guidelines, p. 27, emphasis added).

At several critical junctures, the AI Act thus conflates trustworthiness with the acceptability of risks. That this choice is not free from normative tensions is one of this article's main arguments. Wherever the AI Act leaves discretion for those who apply its rules to determine the acceptability threshold, it requires a normative judgment as to what is acceptable. Who is supposed to make those judgments thus becomes a crucial question which we will revisit soon (see Sections 2.3 and 5.4). Before that, we will shed light on how the AI Act's “risk‐based approach” has important implications for its ability to engineer citizens' actual trust in AI.

### 2.2 Trust, acceptability, and the regulation of risks

Trust is an important topic in risk research (Poortinga & Pidgeon, 2005, p. 199). Risk scholars address the public sector's special role in the reduction of risks based on the states' duty to protect its citizens from harm (Poortinga & Pidgeon, 2005, p. 199). Now, trust in public institutions' ability to effectively regulate technological risks is widely recognized as an important factor of the perceived acceptability of such risks (Freudenburg, 1993; Poortinga & Pidgeon, 2005, p. 199).

This close link between trust, risk regulation, and the perceived acceptability of risks has consequences for the AI Act. Empirical research suggests that trust in institutions is strongly correlated with the perception and acceptability of risks (Poortinga & Pidgeon, 2005, p. 200). There is, however, less clarity on the causal relationships involved. Evidence exists for trust in risk management institutions determining the level of perceived risk and thus the acceptability of risky activities and/or technologies (Eiser et al., 2002; Poortinga & Pidgeon, 2005, p. 200). At the same time, the acceptability of a risk could, instead of being the result, be the determinant of trust (Eiser et al., 2002; Poortinga & Pidgeon, 2005, p. 200). In the latter case, trust and the perception of risk could be the expressions of a more or less favorable attitude (i.e., acceptance) towards a particular technology (Eiser et al., 2002, p. 2425).

For the AI Act as a piece of risk regulation which aims to foster trust in AI, both causal relationships hold important implications. First, if trust in institutions determines the acceptability of AI's risks, then the AI Act itself needs to be trusted to be successful. Second, if the perceived acceptability of risk determines trust, then the actual outcomes of AI and citizens' broader attitude toward the technology will be crucial. Note that citizens' perceptions of a technology and its risks could be vastly different from those of experts. It is the latter, however, who the AI Act tasks with making normative judgments about the acceptability of risks.

The AI Act relies on a complex web of trust relationships which it only implicitly and insufficiently differentiates. Article 3 AI Act defines roles under the AI Act, such as “provider,” “user,” “distributor,” “notified body,” and several public authorities. This taxonomy of AI actors is not clear‐cut. The roles of providers and users can easily become intertwined, for example when the user provides training data to the developer (de Andrade & Zarra, 2022). Moreover, there is no defined “role” for the addressees or subjects of an AI's decision or prediction. Within the limits of our review, these subjects would be the citizens who presumably are expected to trust the AI regulatory system “as a whole.” This raises questions about the model of risk regulation—paternalistic or participatory—chosen by the European Commission.

### 2.3 Paternalistic and participatory elements in risk regulation

There is an ongoing debate in risk regulation about how paternalistically or participatory it should proceed. According to one famous paternalistic take, laypeople and politicians will regularly fail to correctly exercise cost–benefit analyses when it comes to, for example, regulating the risks of a novel technology. Consequently, experts should instead make those assessments and decisions, hence its paternalistic flavor [Kusch, 2007; drawing on the account by Sunstein (2002a, 2002b, 2005)]. One could also call this approach “technocratic.” The alternative model would in either case be a more participatory one, in which non‐experts are included in the assessment of risks and decision‐making about their regulation (Lewens, 2007). Political theory provides normative reasons for why in risk regulation the epistemic asymmetry of laypeople versus experts should not lead to political asymmetry between the two [on epistemic and political asymmetry, see Kusch (2007, p. 149)].

Under the AI Act, AI developers will predominantly assess the acceptability of AI‐specific risks and thus the trustworthiness of AI. They are deemed to be better positioned in terms of expertise than a public authority (Veale & Borgesius, 2021). This will at least be the case until an external AI auditing infrastructure has emerged, able to certify compliance with the AI Act (see also Section 5.4; Mökander et al., 2022). However, for a significant number of high‐risk AI systems, the developers will remain free to choose whether they want to rely on internal controls or involve a third‐party auditor (Mökander et al., 2022). Either way, the AI Act thus largely follows a paternalistic approach.

At the same time, if one takes the AI HLEG's Guidelines into account as well, the seven key requirements for “trustworthy AI” (see Section 2.1) list both procedural as well as substantive elements of trustworthiness. Transparency, for example, can be seen as a procedural requirement which facilitates the involvement of non‐experts to hold AI systems accountable. Other requirements such as technical robustness and safety are more heavily substantive in the sense that they necessarily seem to require technical expertise to deliver particular outcomes. Now, the judgments about the acceptability of risks that the AI Act envisions AI developers to make appear to be substantive, too. The conflation of trustworthiness with the acceptability of risks in the AI Act thus creates a normative tension with the participatory and procedural elements of trustworthiness requirements.

### 2.4 Research questions

Considering the analysis above, the AI Act's understanding of AI's trustworthiness as acceptability of risks combined with its predominantly paternalistic approach to risk regulation invites critical reflection. In this article, we will address two (non‐exhaustive) research questions.

- RQ1: Under what circumstances would the AI Act be able to foster citizens' well‐placed trust in AI?
- RQ2: Are there additional requirements of trustworthiness which have not been properly accounted for by the EU's regulatory approach towards AI?

As outlined in the introduction, our interest lies with AI implemented in the public sector. Answering these questions requires engaging with the complex state of trust research. As a first step of our review, we first propose a prescriptive set of variables to structure the literature.

## 3 A prescriptive set of variables for reviewing trust‐research in the context of AI

As regards AI policy, some have criticized the “over‐simplified rhetoric of trust‐building in the global policy discourse” which “belies the complex and highly contested quality of trust as a concept” (Wilson & van der Velden, 2022). This criticism has merit. A broad variety of definitions of trust can be found within and across academic disciplines including psychology, economics, sociology, political science, law, philosophy, and computer science (Bannister & Connolly, 2011b, p. 139). The field has been described as suffering from “conceptual confusion” and resembling a “conceptual morass” [See the references in McKnight and Chervany (2001, p. 28)]. How to correctly measure trust is the subject of longstanding academic debates [See exemplarily: Beugelsdijk (2006), and the reply by Uslaner (2008); see also Glaeser et al. (2000)]. This conceptual heterogeneity makes it difficult to classify, analyze, and compare results of trust research from different disciplines or even different branches within a given discipline (McKnight & Chervany, 2001, p. 28). The fragmented state of trust research and the incommensurability of trust conceptualizations pose difficulties for any attempt to review and identify coherence and structure within the field.

This section therefore does not aim to provide a singular definition (or typology) of trust and trustworthiness. Instead, it relies on the literature on trust to develop a prescriptive set of variables for analyzing the policy discourse on “trustworthy AI,” including the AI Act. We begin by differentiating the normative and empirical dimensions of the concepts of trustworthiness and trust (Section 3.1). We then address the subjects (Section 3.2), objects (Section 3.3), and roles of trust (Section 2.4).

### 3.1 Normative and empirical dimensions

Within the AI policy discourse, it is helpful to separate normative from empirical uses of the concepts of “trustworthiness” and “trust.” For example, the AI HLEG Guidelines lay out normative key requirements for “trustworthy” AI (see Section 2.1). At the same time, the question of whether someone perceives AI to be trustworthy is an empirical one. When “trust” is used in an empirical sense, it may for example refer to the degree to which someone trusts AI technology. However, there is a normative relation between trust and trustworthiness: if a person or an institution is trustworthy, it could be imprudent (and in rare cases even unjust) to not trust them (Scheman, 2020, p. 28). At the same time, there can be instances of “rational distrust of the apparently trustworthy” (Scheman, 2020, p. 34). For example, historical biases in society may lead affected groups to distrust those who hold credentials of trustworthiness accepted by others (Scheman, 2020, p. 29).^4^

The degrees of trustworthiness and actual trust can thus be misaligned in society. This prompts the normative question of whether people's degree of trust is well‐placed or justified (cf. above in the Introduction). Misalignments may also occur if society begins to trust AI algorithms which are trained with data generated from biased agents and potentially discriminatory practices. Then an AI's discriminatory choices may come to be accepted as more justifiable than when they were made by individuals (Acemoglu, 2021).

The link between a person's tendency to trust and the trustworthiness of the object of trust is likewise reflected in empirical trust research (Mayer et al., 1995). Trust is improbable to be produced on demand (Cook & Santana, 2020), and impossible to achieve on command. Trustworthiness, on the other side, can be institutionally enforced, for example through contracts or audits with the threat of sanction (Hardin, 2002). Regulations such as the AI Act are an attempt at an institutional enforcement of trustworthiness. Enhancing trustworthiness can increase levels of trust if the increase in trustworthiness is recognized in the population (Hardin, 2002).

Such “trust engineering” does, however, not always achieve its goal and may even backfire. For example, careful communication with the public has been argued by some to be conducive to strengthening public trust (Shah, 2018). Yet when the UK mandated annual reports to Parliament on the detailed use of animals in scientific laboratories as an effort to increase trustworthiness through transparency, this seemingly only increased public mistrust in scientists and the pharmaceutical industry (O'Neill, 2002). There is further research which suggests that transparency can lower trust in government (See the references in Bannister & Connolly, 2011a). Transparency, as we will show further below, has a demanding relationship with raising trust levels (de Bruijn et al., 2022, p. 4). This is not to say, however, that transparency as a normative requirement for trustworthiness is thus misguided. It merely shows the difficulties with engineering trust and moving from a normative perspective to an empirical evaluation.

### 3.2 Subjects of trust

The next key variable for reviewing the trust literature is the subject of trust (often called the “trustor”). In this article, we are primarily interested in the trust of citizens, that is, human trust. Other relevant human agents would be public servants, who will likely be the primary users of AI systems in public institutions. Note that in certain areas of the literature the trustor may also be a computer or artificial agent, for example in information security and blockchain technology [See exemplarily: Yadav and Kushwaha (2021)].

### 3.3 Objects of trust

As Nissenbaum writes, one can “trust (or distrust) persons, institutions, governments, information, testimony, deities, physical things, systems, and more” (Nissenbaum, 1999). Below, we will provide a three‐set typology of objects of trust (also called “trustees”) for our interest in AI policy: people, institutions, and technology. When a public institution decides to use AI, citizens' trust in this operation may be comprised of their trust in the particular institution in question, institutions in general, the public servant(s) which handles or “owns” the AI (if there is one), the technology itself, and in their fellow citizens. These are to a large degree interacting societal processes (Robinson, 2020, p. 2).

#### 3.3.1 People

As regards the concept of “trust,” it is common to differentiate “interpersonal trust” from “institutional trust.” Through interpersonal trust, one trusts other people either personally, for example through personal knowledge, or through their attributes, such as an expert's credentials (McKnight & Chervany, 2001, p. 37). One can further differentiate the trust in persons as either dispositional when trusting people in general (also called “generalized” or “social trust”) or as interpersonal in the stricter sense, when trust is directed at another specific individual (McKnight & Chervany, 2001, p. 40).

#### 3.3.2 Institutions

“Institutional trust,” on the other hand, is sometimes defined as trust in institutions (or sometimes as “legitimacy”), such as government institutions or public institutions (Kavanagh et al., 2020; see the references in Robinson, 2020, p. 3). Interpersonal trust and institutional trust are also interacting societal processes. Empirical research suggests that trust in state institutions has a positive impact on trust between people, including strangers (Sønderskov & Dinesen, 2016; Spadaro et al., 2020). Institutional trust is also used to describe a societal evolution: from earlier‐stage societies with local, interpersonal trust (for example between family members) to modern complex societies in which strangers can now be trusted because “licensing or auditing or law or governmental enforcement bodies [are] in place to make sure the other person was either afraid to harm them or punished if they did harm them.” (McKnight & Chervany, 2001, p. 37; drawing on Zucker, 1986). Such trust‐enhancing, impersonal features enable people to trust each other without knowing one another personally. This is paramount in modern societies in which trust often cannot be based on prior social interactions or reputational information shared by others (Cook & Santana, 2020, p. 198; Spadaro et al., 2020, p. 2; citing, Zucker, 1986; for a state‐engineered move from kinship‐based trust to institutional trust, see Zou, 2021). In this view, institutions such as governments or expert systems function as facilitators of the emergence of trust (Cook & Santana, 2020, p. 190).

Institutional trust has often been described as laying the foundation for the relationship and interaction between laypeople and experts (Chen & Wen, 2021; drawing on Camporesi et al., 2017; Giddens, 1990). Note that laws and legal systems (contracts, property rights, regulations such as the AI Act, etc.) can also be regarded as institutions. This is common in economic literature which focuses on economic growth as an outcome of institutional trust (See the references in Hwang, 2017).

Institutional trust can thus refer to both trust in institutions as well as institutions as a facilitator of the emergence of trust. If citizens say that they do not trust the public sector to utilize AI‐based decision‐making, then this could mean that they express a concern about personal risk, that is, they are not willing to expose themselves to a position of vulnerability vis‐á‐vis the public institution. While the willingness to be vulnerable in view of risk and uncertainty about the trustee's intentions and actions is a common definition of trust, citizens could also refer to something else (Bannister & Connolly, 2011b, p. 139). They could mean that they do not trust the government to be competent enough to properly use AI or to make “right” or “fair” decisions with it (Cf., albeit without the focus on AI: Bannister & Connolly, 2011b, p. 139).

Recently, some trust scholars have argued that with technologies such as blockchain, “distributed” trust is beginning to replace institutional trust as the defining mode of creating trust in today's societies (Botsman, 2017). Because of its decentralized architecture, some argue that blockchain trust is different from other types of trust (for an overview, see Werbach, 2018). From the point of view of this article, however, the budding literature on blockchain and trust concerns technology as an object of trust.

#### 3.3.3 Technology

Trust in (digital) technology has been studied extensively in social science as well as in computer science, information science, and communication research (for the latter, see the references in Bodó, 2021, p. 2680). When a public institution begins to implement novel AI systems, this can change its perceived trustworthiness and the processes by which it produces trust in its operations (Bodó, 2021, p. 2675). As Bodó writes, the use of digital technologies in institutions “creates new uncertainties, conflicts of interest, and modes of operation; it restructures values and ethics” (Bodó, 2021, p. 2675).

At the same time, technology is itself deployed to produce trust. Machine learning, for example, is used in systems that seek to create trust from quantitative insights. Search platforms rank results according to relevance and social media platforms (more or less) thoroughly filter out untrustworthy information (Bodó, 2021, p. 2679; referring to Tang & Huan, 2015). Platforms such as Airbnb, Taskrabbit, and Uber have arguably increased trust in strangers to provide services. Some argue they have reigned in a new era of “distributed trust” (Botsman, 2017). In the literature, there is, however, also long‐standing skepticism as to whether technology can produce trust (Nissenbaum, 1999; Zou, 2021), especially towards government institutions (Bannister & Connolly, 2011b). Normative constraints (such as limits on the social scoring of citizens in Article 5(1)(c) AI Act) and value systems will predictably shape the rollout of any such trust‐producing technology.

### 3.4 Roles of trust

Lastly, another important variable for reviewing trust research is the role of trust. In the empirical literature, this issue regularly comes up in debates about whether revealed correlations represent causal relations and if so, in which direction causality flows (van Ingen & Bekkers, 2015, p. 277). Mutual effects can be plausible (see the relationship between trust and the perceived acceptability of risk in section 2.2).

In empirical research, the role of trust can be either be the “dependent variable” (i.e., the outcome variable) or the “independent variable” (i.e., the explanatory variable; on these statistical terms, see Upton & Cook, 2014). Simplifying for the sake of comparability, in normative papers trust can similarly play the role of a “consequent” or an “antecedent.” To represent the same set of criteria for both empirical and normative papers in the next section, we label the conditionality of trust as the role of independent/dependent variable (for empirical papers) and antecedent/consequent (for normative papers). Of course, normative papers do not set out to causally explain the emergence of trust. Nonetheless, normative papers often feature an argumentative structure of the type that “to be trustworthy, AI needs to be transparent.” Here, transparency is seen as a necessary (not necessarily sufficient) condition for trustworthiness, that is, an antecedent of trust. Vice versa, an argumentative structure of the type “for courts to rely on AI in sentencing, it needs to be trustworthy” in which trustworthiness is the necessary (not necessarily sufficient) condition for useability was labeled as trust's role as an “antecedent.”

To introduce some clarity to the broad and diverse field of research on trust, AI, and the public sector, we limit our review to the following values in the variables (see Table 1): that the trustor is the citizen (as opposed to a public servant using the AI); that the object of trust is a public institution (as opposed to individuals, public servants or generalized trust amongst individuals in a society) or that trust in AI in the public sector is supposed to be strengthened via institutional trust (such as laws and regulations); and that the role of trust is the “dependent variable” (as opposed to the “independent variable”) or “consequent” (as opposed to an “antecedent”).

**TABLE 1:** Variables for trust review

Trustor	
Citizen	✓
Public servant	
Object of trust	
Institution	✓
Individual(s)	
Technology	
Role of trust	
Independent variable	✓
Dependent variable	
Study type	
Normative	✓
Empirical	✓

## 4 Review: Trust and trustworthy AI in the public sector

We now apply these trust‐review variables to conduct a narrative review for AI in the public sector. We begin our review by laying out our review method (Section 4.1). We then review the normative (Section 4.2) and empirical (Section 4.3) literature, respectively.^5^

### 4.1 Method

The review proceeded in three steps: (1) identifying relevant literature in research databases; (2) screening the results to produce an initial sample for closer inspection and applying criteria of inclusion and exclusion to the initial sample to produce a final sample, divided between normative papers and empirical papers, and (3) analyzing the final sample.^6^

The initial search was implemented on 20 April 2022 on SCOPUS and Web of Science, two major international and multidisciplinary research databases. The following string of search terms was applied: (TITLE‐ABS‐KEY (“artificial intelligence” OR “machine learning” OR algorithm* OR “automated decision‐making”)) AND (TITLE‐ABS‐KEY (“public administration” OR “public sector” OR “government” OR “public policy” OR “public service*”)) AND (TITLE‐ABS‐KEY (trust OR trustworthiness OR trustworthy)).^7^ The terms of AI selected follow a previous scope analysis (Pham et al., 2014).

The search returned 466 results on SCOPUS and 203 results on Web of Science. After using Zotero to eliminate duplicates, the search results identified 541 unique papers.

After screening the title, keywords, and abstracts of the sample of *n* = 541 search results, using the inclusion and exclusion criteria listed below in Table 1, an initial sample of *n* = 109 papers remained (Table 2).^8^

**TABLE 2:** Inclusion and exclusion criteria

*Inclusion criteria*
(1) Academic or commercial research
(2) Studies published in English
(3) Focus on the public sector
(4) Presence of terms related to AI in title/abstract/keywords
(5) Mentions “trust” in title/abstract/keywords
*Exclusion criteria*
(1) Focus on the private sector or not specific to the public sector
(2) Focus only on technical aspects
(3) Studies focused on forms of trust other than institutional trust

We then subjected the initial sample to closer inspection, now including the main text, using the same inclusion and exclusion criteria. The final sample consisted of 71 papers. We divided this sample into two, differentiating papers exclusively as predominantly normative (*n* = 38) or empirical (*n* = 33). We interpreted the terms “normative” and “empirical” rather inclusively based on the paper's contents and its main contribution. For example, papers providing an overview of existing ethical and legal frameworks for AI governance were categorized as normative. Papers developing a theoretical model for explaining the emergence of trust without, however, empirically testing it were classified as empirical (Table 3).

**TABLE 3:** Samples of papers

Sample	Number of papers
Search results	541
Initial sample	109
Final sample	71
Normative sample	38
Empirical sample	33

### 4.2 Analysis of normative papers

We analyzed the batch of normative papers (*n* = 38) in the final sample along three variables: that the subject of trust is the citizen (as opposed to a public servant using the AI); that the object of trust is a public institution (as opposed to individuals, public servants or generalized trust amongst individuals in a society) or that trust in AI in the public sector is supposed to be strengthened via institutional trust (such as laws and regulations); and the role of trust is the “consequent” (as opposed to an “antecedent”). To reiterate, we developed the variables of subject, object, and role of trust in Section 3 considering the difficulties of reviewing trust research. Sorting the literature along these variables allows us to narrow the sample of papers further down to those which are comparable to the largest degree possible. As mentioned in Section 3, a high correlation between various types of trust—for example, generalized trust in individuals may increase trust in institutions—is nevertheless to be expected (Table 4).

**TABLE 4:** Sample of normative papers

			Trustor		Object of trust			Role of trust	
Year	Author	Title	Citizen	Public servant	Institution	Individual(s)	Technology	Ind Var	Dep Var
2022	de Bruijn, H.; et al.	The perils and pitfalls of explainable AI	+				+		+
2022	Lee, S.S.	Philosophical evaluation of the conceptualization of trust in the NHS' Code of Conduct for artificial intelligence‐driven technology	+				+		+
2022	Wilson, C.; van der Velden, M.	Sustainable AI	+		+		+	+	+
2021	Shank, C.E.	Credibility of Soft Law for Artificial Intelligence‐Planning and Stakeholder Considerations			+				+
2021	Roski, J.; et al.	Enhancing trust in AI through industry self‐governance	+	+	+	+	+		+
2021	Mökander, J.; Floridi, L.	Ethics‐Based Auditing to Develop Trustworthy AI			+				+
2021	Pickering, B.	Trust, but verify	+		+				+
2021	Gerdes, A.	AI can turn the clock back before we know it	+		+		+		+
2021	Li, G.	State Control by Stealth in the Big Data Era—From WeChat to the Social Credit System in China	+		+		+		+
2021	Drake, A. et al.	Legal contestation of artificial intelligence‐related decision‐making in the United Kingdom	+				+	+	+
2021	Medaglia, R.; et al.	Artificial Intelligence in Government				+	+		
2021	Smidt, H.J.; Jokonya, O.	The challenge of privacy and security when using technology to track people in times of COVID‐19 pandemic	+		+				+
2021	Zou, S.	Disenchanting trust: Instrumental reason, algorithmic governance, and China's emerging social credit system	+			+			+
2021	Anna, Z.; Vladimir, E.	State regulation of the IoT in the Russian Federation					+	+	
2020	Mollura, D.I. et al.	Artificial intelligence in low‐ and middle‐income countries	+				+	+	
2020	Robinson, S.C.	Trust, transparency, and openness	+		+	+	+	+	+
2020	Shneiderman, B.	Bridging the gap between ethics and practice			+		+		+
2020	Madhavan, R.; et al.	Toward Trustworthy and Responsible Artificial Intelligence Policy Development					+		+
2020	Kuziemski, M.; Misuraca, G.	AI governance in the public sector	+		+				+
2020	Vesnic‐Alujevic, L.; et al.	Societal and ethical impacts of artificial intelligence					+		
2020	Carter, D.	Regulation and ethics in artificial intelligence and machine learning technologies					+		
2020	Carter, S.M.; et al.	The ethical, legal, and social implications of using artificial intelligence systems in breast cancer care	+		+	+			+
2020	Kitsos, P.	The limits of government surveillance	+		+				+
2020	Katulić, T.	Towards the trustworthy AI	+				+		+
2020	NíFhaoláin, L.; et al.	Assessing the appetite for trustworthiness and the regulation of artificial intelligence in Europe					+		
2020	Harrison, T.M.; Luna‐Reyes, L.F.	Cultivating Trustworthy Artificial Intelligence in Digital Government	+		+		+		+
2020	Ruttkamp‐Bloem, E.	The Quest for Actionable AI Ethics				+	+		+
2020	Rêgo de Almeida, P.G.; et al.	Artificial intelligence regulation					+		
2020	Rees, C.	The Ethics of Artificial Intelligence	+				+		+
2019	Gilbert, G.L.; et al.	Communicable Disease Surveillance Ethics in the Age of Big Data and New Technology	+		+	+	+		+
2019	Guan, J.	Artificial Intelligence in Healthcare and Medicine				+			+
2019	Paic, A.	Policies for artificial intelligence in science and innovation					+		
2018	Liu, X.; Reisenbichler, G.V.	Trees of knowledge	+				+		+
2018	Shah, H.	Algorithmic accountability	+		+		+		+
2020	Land, MK; Aronson, JD	Human Rights and Technology	+		+				
2021	Young, MM; et al.	Artificial Intelligence and Administrative Evil			+				
2021	Manjarres, A; et al.	AI4Eq					+		
2020	Nesterova, I	Mass data gathering and surveillance					+		

Out of this batch, we ended up with 13 normative papers which had positive values in all three variables (Table 5). Below, we discuss these papers along with common themes we identified in their findings.

**TABLE 5:** Sample of reviewed normative papers

			Trustor		Object of Trust			Role of Trust	
Year	Author	Title	Citizen	Public Servant	Institution	Individual(S)	Technology	Ind Var	Dep Var
2022	Wilson, C.; van der Velden, M.	Sustainable AI	+		+		+	+	+
2021	Roski, J.; et al.	Enhancing trust in AI through industry self‐governance	+	+	+	+	+		+
2021	Pickering, B.	Trust, but verify	+		+				+
2021	Gerdes, A.	AI can turn the clock back before we know it	+		+		+		+
2021	Smidt, H.J.; Jokonya, O.	The challenge of privacy and security when using technology to track people in times of COVID‐19 pandemic	+		+				+
2021	Li, G.	State Control by Stealth in the Big Data Era—From WeChat to the Social Credit System in China	+		+		+		+
2020	Robinson, S.C.	Trust, transparency, and openness	+		+	+	+	+	+
2020	Kuziemski, M.; Misuraca, G.	AI governance in the public sector	+		+				+
2020	Carter, S.M.; et al.	The ethical, legal, and social implications of using artificial intelligence systems in breast cancer care	+		+	+			+
2020	Kitsos, P.	The limits of government surveillance	+		+				+
2020	Harrison, T.M.; Luna‐Reyes, L.F.	Cultivating Trustworthy Artificial Intelligence in Digital Government	+		+		+		+
2019	Gilbert, G.L.; et al.	Communicable Disease Surveillance Ethics in the Age of Big Data and New Technology	+		+	+	+		+
2018	Shah, H.	Algorithmic accountability	+		+		+		+

#### 4.2.1 (Public) Accountability

Accountability is one of the most salient conditions for trustworthiness of AI in the public sector mentioned in the normative literature. Understood in a broader sense, accountability includes a long list of principles and mechanisms. Neither principle nor mechanism is on its own sufficient and thus requires being combined with others (Shah, 2018, p. 2). Merging the elements of accountability from the papers provides the following (inconclusive) list: transparency (including the sharing of incidents), algorithmic impact assessments, screening for bias, monitoring outcomes for differential impacts (not least in disadvantaged communities), explainability of decisions (including counterfactual explanations), (informed) consent, rights to challenge and redress automated decision‐making, an independent tribunal or ombudsman, good AI governance, human actors in the decision‐loop, data management frameworks, data literacy of public officials, participatory AI development and testing, and fairness (Harrison & Luna‐Reyes, 2020; Kitsos, 2020; Li, 2021; Robinson, 2020, p. 10; Shah, 2018, pp. 2–3; Smidt & Jokonya, 2021; Wilson & van der Velden, 2022, p. 7).

The reviewed normative literature does not merely produce a “wish‐list” of accountability requirements. The papers themselves anticipate obstacles both to their meaningful implementation and their ability to successfully raise trust levels amongst citizens. These obstacles are often drawn from empirical research with ambiguous results.

Robinson, for example, restates empirical findings which seem to both underscore the importance of transparency for creating trustworthy governments (Robinson, 2020, p. 3; drawing on Grimmelikhuijsen et al., 2013) and obtained a negative result after testing the effect of transparency on trust (Robinson, 2020, p. 10; citing Schmidt et al., 2020). Shah likewise states that for machine learning models, transparency may be of limited value and not even be key to accountability [Shah, 2018, p. 2; drawing on philosophical work such as O'Neill's (see Section 3.1)]. Similarly, de Bruijn et al. emphasize the difficult relationship between explainability and transparency on the one side and trust on the other side (de Bruijn et al., 2022, p. 4; citing Auger, 2014; drawing also on the work of Bannister & Connolly, 2011a). The authors consider that the effect of explainable AI on trust levels may be limited depending on societal context. AI systems with high impact and operating in a highly politicized environment, even if well‐explainable are likely to generate conflict. Stakeholders' views will differ as to what the “right” or “fair” decision is. This increases the chances that the public will distrust the explanation given for an AI's decision (de Bruijn et al., 2022, p. 4; on the contextuality of trust in institutions and their use of technology, see also Bannister & Connolly, 2011a).

One should not, however, conclude that the normative desirability of transparency and explainability is easily tainted by the existence of knowledge asymmetries. After all, attributing responsibility for decisions based on an AI's recommendations is a highly complex problem (See also, Carter et al. (2020). See also, Bannister & Connolly, 2011a). It becomes nearly impossible without transparent and intelligible procedures (see further, Section 4.2.7).

De Bruijn et al. further consider that explainable AI may not lead to trust and technology uptake if people “who do not know how AI works” do not have enough confidence in it (de Bruijn et al., 2022, p. 4). This points to a second obstacle for a successful implementation of accountability measures, namely the public's (alleged) lack of technical expertise. Knowledge asymmetries are not only stated as an obstacle to success but also as a normative concern. They challenge participatory models of trustworthiness and regulating AI's risks (Robinson, 2020; Shah, 2018; see also, Wilson & van der Velden, 2022, p. 7; Carter et al., 2020, p. 30). For example, commenting on a Finnish AI policy Robinson criticizes its “downplay” of non‐expert citizens' concerns about transparency in AI, as it “assumes that only individuals of high technical understanding can appreciate the complexity of systems,” potentially eroding citizen's trust (Robinson, 2020, p. 11). Furthermore, while knowledge asymmetries are not exclusive to AI and exist for other technologies as well, the ongoing general public debate about the dangers of AI is expected to require some *public* accountability about its usage (Carter et al., 2020, p. 30).

The potentially transformative effects of AI used within public institutions have been associated with the different reasoning style of AI decision‐making when compared to human judgment processes (Harrison & Luna‐Reyes, 2020, pp. 498–501). Without public trust in AI, government decision‐making based on the technology may be found to lack legitimacy (Harrison & Luna‐Reyes, 2020, p. 495). As a remedy, Harrison and Luna‐Reyes suggest the participatory development and testing of AI, including the interaction between stakeholders such as “software developers,” “domain experts,” “experts and users,” and “users and others affected by system outcomes” (Harrison & Luna‐Reyes, 2020, p. 505). While including users and those affected by AI's decision‐making, the paper does not clarify how the feedback from non‐experts can be utilized so that it increases public trust which is actually justifiable. The authors point to a lack in the literature, as at their time of writing there was little empirical research on how to best implement a participatory model for AI governance (Harrison & Luna‐Reyes, 2020, p. 506).^9^

#### 4.2.2 Mediated trust

The reviewed normative literature connects the lack of AI expertise ascribed to “ordinary” citizens with the remedial notion of “proxy trust” or “mediated trust” (Bodó, 2021, p. 2669; Wilson & van der Velden, 2022, p. 7). Individuals' trust toward an AI system will regularly be established via proxies (Wilson & van der Velden, 2022, p. 7). Wilson and van der Velden recognize such proxy trust in a varied set of objects of trust. First, they explicitly mention what Bodó calls “trust mediators,” that is, technologies such as platforms and sharing services (instead of institutions or persons) which aim to produce trust (see Section 3.3.3; Bodó, 2021, p. 2669; Cf: Wilson & van der Velden, 2022, p. 7). Second, the authors distinguish “implicit” trust which is “invested in the larger system of public and private actors that are associated with the AI at issue.” (Wilson & van der Velden, 2022, p. 7; drawing on Steedman et al., 2020). Such implicit trust will regularly be shaped via institutions, as the authors emphasize that public trust in AI systems must not be “blind, but appropriate” (Wilson & van der Velden, 2022, p. 7) while problematizing the knowledge asymmetries for participatory models of trust‐building (Such as those the authors find in Wirtz et al., 2020). This implies a focus on conditions for trustworthiness—such as the accountability mechanisms listed under Section 4.2.1—instead of levels of actual trust.^10^

#### 4.2.3 Consent

Some suggest facilitating informed consent to foster trust in AI in public services (Wilson & van der Velden, 2022, p. 7). While consenting to AI‐based decision‐making should be mostly uncontroversial in its normative desirability, it faces two problems. First, as mentioned above a government's use of AI can include elements of coercion. If an AI system is rolled out, for example, in law enforcement or public welfare without keeping non‐AI‐based systems running and available as an alternative option, consent is de facto meaningless. Second, consent suffers from knowledge asymmetries in similar ways as transparency and explainability do.

Pickering therefore challenges the use of informed consent (Pickering, 2021). He develops an alternative conception of “trust‐based consent” starting from Eyal's observation that informed consent does not automatically promote trust in medical care (Pickering, 2021, p. 10; drawing on Eyal, 2014). Instead, Pickering views trust as a “constant negotiation” between a trustor's willingness to trust and the trustee's display of indicators of trustworthiness (Pickering, 2021, p. 11). Drawing on work in the domain of management and organizational studies, Pickering offers a behavioral approach to trust and consent: trustees must find ways to continuously demonstrate their trustworthiness indicators to the trustor (Pickering, 2021, pp. 10–11; drawing on Mayer et al., 1995).

#### 4.2.4 Modes of regulation

In the normative literature, different types of regulatory measures are being suggested, spanning from industry self‐regulation to ethical and decidedly legal frameworks. As regards the law, the domains of data protection law, non‐discrimination law, and sector‐specific considerations are held to be of major importance (Shah, 2018, p. 3). Beyond regulation, Shah suggests three further measures which could improve trustworthiness: diversity in the technology workforce, ethics training and codes of conduct for the profession of data scientists, and “more reflective work” through independent deliberative bodies such as the UK's Ada Lovelace Institute, which can help to deliver ethics for data science and to set standards (Shah, 2018, p. 4).

Roski et al. suggest industry self‐governance which incorporates “multistakeholder” participation as a means to counter a stated “growing mistrust of AI solutions” in the health care sector and to mitigate risks (Roski et al., 2021; another argument in favor of “soft‐law” is presented by Shank, 2021). As regards its operationalization, the authors also consider certification and accreditation programs (Roski et al., 2021, p. 1585). Gilbert et al. claim that an ethical framework is necessary to “optimise benefits and minimise risks, protect vulnerable populations and build public trust” (Gilbert et al., 2019). At the same time, others argue in favor of legal regulation, as the reliance on voluntary standards and self‐governance is “disregarding power‐related considerations” (Kuziemski & Misuraca, 2020, p. 10).

#### 4.2.5 Communication of policies and their failures

Closely related to the principle of transparency is the stated importance of public communication around governments' implementation of AI, including its failures. Poorly implemented and poorly communicated policies are said to “quickly erode public trust and impact perceptions of government transparency” (Robinson, 2020, p. 11). At the same time, others note the risk of an undue loss of trust in technology if stakeholders who initially held AI systems to be trustworthy, distrust even largely reliable applications after observing errors (Kuziemski & Misuraca, 2020, p. 4; citing Dzindolet et al., 2003). There is thus a risk that insufficiently developed AI are adopted too early, thus putting trust in the entire system at risk (Kuziemski & Misuraca, 2020, p. 4). Robinson adds how easily trust can be eroded in digital interactions through “hacking, fraud, or technological incompetence on the part of industry or government institutions” (Robinson, 2020, p. 2). He argues that government institutions must maintain high standards of information security (Robinson, 2020; drawing on Ministry of Finance, Public Governance Department, 2019). Relatedly, Gilbert et al. discuss the potential of Big Data‐based communicable disease surveillance for public health (Gilbert et al., 2019). The authors identify the risk that “inaccurate or exaggerated outbreak predictions or modelling” could lead to “unnecessary public fear and loss of trust in public health authorities” (Gilbert et al., 2019, p. 183). They thus suggest public education and consultation initiatives, supported by privacy regulation and standards (Gilbert et al., 2019, p. 183).

As a remedy, Kuziemski and Misuraca suggest a deliberative model of participatory AI governance. The authors state the necessity of conversations about the ends and means of using AI in the public sector, including the populations at stake (Kuziemski & Misuraca, 2020, p. 10). However, evaluating the potential impact of the use of AI in the public sector faces a benchmark problem. When assessing public sector innovation, the market will often be absent and outcome monitoring has thus historically relied on self‐reported measures such as interviews and surveys (Kuziemski & Misuraca, 2020, p. 10).

#### 4.2.6 Political economy

Lastly, common amongst several reviewed normative papers are concerns about the market structures of the data economy (and hence AI research). Shah considers the growth of private companies to become “data monopolies” and the impact of data technologies on democracy (Shah, 2018, pp. 4–5). Likewise, Gerdes warns that societal trust in science may decline because AI research is influenced by “corporate interests,” hence putting the “credibility of research […] at risk” (Gerdes et al., 2021). The normative literature also engages in more visionary thinking about the future of AI. Shah states the need for institutions which enable a “shared vision of what sort of algorithmic society we are seeking to build,” and for moving from ethics as “negative screening” to thinking how AI can serve the public interest (Shah, 2018, pp. 4–5). Relatedly, Wilson and van der Velden list trust as one of several boundary conditions of socially “sustainable” AI (Lane, 2014).

#### 4.2.7 Interim findings

In response to RQ1, the reviewed normative literature appears to prefer a more participatory model of establishing trustworthiness in public sector AI systems. This preference is visible in the importance attached to public accountability, informed consent, and the public communication of the use and failures of AI. This creates some tension with the expertocratic model of risk regulation pursued in the AI Act (see Section 2.3).

Nevertheless, there appears to be consensus in the literature that well‐placed trust in AI systems in the public sector requires institutions of public accountability. Participation, however, faces knowledge asymmetries between laypeople citizens and AI developers/users. The suggestion to develop a behavioral notion of “trust‐based” consent resembles approaches in the philosophical literature which aim to address knowledge asymmetries between experts and laypeople. Instead of first‐order assessments of expertise, laypeople could instead engage in second‐order assessments. For example, they could assess experts' behavior, including their rationality in conversation and willingness to accept external peer review (Lane, 2014). This point relates to the AI Act's intended system of oversight and internal or external audits (see Section 5.4).

Another complicating factor lies in the normative literature's appreciation of empirical observations that certain accountability mechanisms such as transparency and explainability do not necessarily lead to higher levels of actual trust (see Section 4.3.2). Such empirical findings do not taint the normative desirability of transparency and explainability. They merely illustrate difficulties for policy makers when aiming to engineer trust. It would further be a grave empirical fallacy to conclude that because principles such as transparency may fail to produce trust, non‐transparency will therefore produce it. Moreover, actual trust in an AI‐supported public decision‐making system will regularly be an amalgam of all the different objects of trust involved, including people, institutions, and technology (cf. Section 3.3; See also, Robinson, 2020, p. 2).

The normative literature's variety in suggested modes of regulation—stemming from law over ethics to industry self‐regulation—highlights the risk of “framework shopping.” Without legal regulation such as through the proposed AI Act, developers of AI systems remain free to select the normative framework for “trustworthy AI” which best fits their commercial interests. The political economy of data that the literature addresses, however, falls outside of the risk‐centered remit of the AI Act proposal.

As regards, RQ2, knowledge asymmetries can indeed motivate an additional requirement of trustworthiness, namely intermediaries which are themselves trustworthy. The notions of “trust proxies” or “mediated trust” mentioned in the literature point in this direction. In Section 5.4, we outline further how intermediary institutions can help to create well‐placed trust.

### 4.3 Analysis of empirical papers

We analyzed the batch of empirical papers (*n* = 33) in the final sample (Table 6) along the same three variables: that the trustor is the citizen, that the object of trust is at least also institutional trust, and that the role of trust is the dependent variable. Our interest here lies with the empirical antecedents of institutional trust as regards the use of AI in public institutions.

**TABLE 6:** Sample of empirical papers

			Trustor		Object of trust			Role of trust	
Year	Author	Title	Citizen	Public Servant	Institution	Individual(S)	Technology	Ind Var	Dep Var
2022	Yalcin, G.; et al.	Perceptions of Justice By Algorithms	+		+	+	+		+
2022	Kennedy, R.P.; et al.	Trust in Public Policy Algorithms	+	+	+	+	+		+
2022	Bélisle‐Pipon, J.‐C.; et al.	Artificial intelligence ethics has a black box problem							
2021	Ploug, T.; et al.	Population Preferences for Performance and Explainability of Artificial Intelligence in Health Care	+		+	+	+	+	
2021	Wang, C.; et al.	Public and private value creation using artificial intelligence	+		+		+		+
2021	Kumar, S.; et al.	Mapping the barriers of AI implementations in the public distribution system		+			+	+	
2021	Sangers, T.E.; et al.	Views on mobile health apps for skin cancer screening in the general population	+				+	+	
2021	Chen, T.; et al.	AI‐based self‐service technology in public service delivery	+		+			+	
2021	Spatola, N.; MacDorman, K.F.	Why Real Citizens Would Turn to Artificial Leaders	+		+	+		+	
2021	Belton, O.; Dillon, S.	Futures of autonomous flight	+		+	+		+	
2021	Karatzogianni, A.	Research design for an integrated Artificial Intelligence ethical framework					+		
2021	Ingrams, A.; et al.	In AI we trust?	+			+	+		+
2021	Drobotowicz, K.; et al.	Trustworthy AI Services in the Public Sector	+				+		+
2021	Sarea, A.; et al.	Evaluation of Compatibility of Cloud‐based Applications, Credibility, and Trust Perceptions on the Adoption of Cloud Technology	+			+	+	+	+
2021	Aoki, N.	The importance of the assurance that “humans are still in the decision loop” for public trust in artificial intelligence	+		+		+		+
2021	Chen, Y.‐N.K.; Wen, C.‐H.R.	Impacts of Attitudes Toward Government and Corporations on Public Trust in Artificial Intelligence	+		+		+	+	+
2020	Aoki, N.	An experimental study of public trust in AI chatbots in the public sector	+		+		+		+
2020	Atluri, V.; et al.	Security, privacy and trust for responsible innovations and governance			+	+			
2020	Hashim, H.S.; Al Sulami, Z.A.	Cloud computing‐based e‐government in Iraq using partial least square algorithm		+	+		+	+	
2020	Degeling, C. et al.	Community perspectives on the benefits and risks of technologically enhanced communicable disease surveillance systems	+		+			+	
2020	Zhang, B.; Dafoe, A.	U.S. Public opinion on the governance of artificial intelligence	+		+				+
2020	Flügge, A.A.; et al.	Algorithmic decision making in public services	+	+	+		+		+
2020	Kuberkar, S.; Singhal, T.K.	Factors influencing adoption intention of ai powered chatbot for public transport services within a smart city	+				+		+
2019	Ranerup, A.; Henriksen, H.Z.	Value positions viewed through the lens of automated decision‐making		+		+			+
2019	Cho, S.H.; et al.	A Study on the Factors Affecting the Continuous Use of E‐Government Services	+	+	+		+	+	+
2018	Al‐Sulami, Z.A.; Hashim, H.S.	Measuring the success of e‐government systems	+		+		+	+	
2016	Drew, C.	Data science ethics in government	+		+				+
2014	Fu, K.‐J.; Lee, C.‐P.	The role of trust in the prioritization of channel choices	+		+		+	+	
2011	Ek Styvén, M.; et al.	“IT's complicated…”	+		+		+	+	
2010	Wang, T.; Lu, Y.	Determinants of trust in e‐government	+		+		+		+
2007	Oduor, K.F.; Campbell, C.S.	Deciding when to trust automation in a policy‐based city management game					+		+
2021	Miller, SM; Keiser, LR	Representative Bureaucracy and Attitudes Toward Automated Decision Making	+		+	+		+	
2020	Al Bajjari, F; Hassanpour, R	A Model for Adopting Cloud Computing in Government Sector					+	+	

Out of this batch, we ended up with nine empirical papers with positive values in all three variables (see Table 7). Again, we discuss common themes in this literature.

**TABLE 7:** Sample of reviewed empirical papers

			Trustor		Object of trust			Role of trust	
Year	Author	Title	Citizen	Public Servant	Institution	Individual(s)	Technology	Ind Var	Dep Var
2022	Yalcin, G.; et al.	Perceptions of Justice By Algorithms	+		+	+	+		+
2022	Kennedy, R.P.; et al.	Trust in Public Policy Algorithms	+	+	+	+	+		+
2021	Wang, C.; et al.	Public and private value creation using artificial intelligence	+		+		+		+
2021	Aoki, N.	The importance of the assurance that “humans are still in the decision loop” for public trust in artificial intelligence	+		+		+		+
2020	Aoki, N.	An experimental study of public trust in AI chatbots in the public sector	+		+		+		+
2020	Zhang, B.; Dafoe, A.	U.S. Public opinion on the governance of artificial intelligence	+		+				+
2019	Cho, S.H.; et al.	A Study on the Factors Affecting the Continuous Use of E‐Government Services	+	+	+		+	+	+
2016	Drew, C.	Data science ethics in government	+		+				+
2010	Wang, T.; Lu, Y.	Determinants of trust in e‐government	+		+		+		+

Before we delve into the analysis, some preliminary remarks are due. First, trust in computers in general and in AI, in particular, features a broad variety of research approaches observable in the initial sample, including the “human‐computer‐trust scale” (Oduor & Campbell, 2007); attachment theory (Spatola & MacDorman, 2021); social psychology theory which measures perceptions of competence, benevolence, and honesty (Ingrams et al., 2021; drawing on Grimmelikhuijsen et al., 2013, p. 138; and behavioral reasoning theory, Gesk & Leyer, 2022, p. 2), to name just a few. As mentioned, this diversity of approaches makes the comparison of results across the literature difficult, even more so than in the normative batch of papers.

Second, initial research on the acceptance of AI in the public sector suggests that AI is perceived differently from existing software systems, not least because of its recognized ability to circumstantially adapt its behavior (Gesk & Leyer, 2022, p. 3). Moreover, trust in public institutions is closely linked to their perceived legitimacy. Automated systems as the sole decision‐maker have been found to be perceived as illegitimate (Starke & Lünich, 2020). These issues resonate in the empirical batch of the final sample.

Lastly, the results in these empirical studies are not generalizable (Cf. Aoki, 2021, pp. 6–7; Aoki, 2020). The findings cannot readily be applied to other times, places, and contexts. Not a single study in the empirical batch had EU citizens as participants.

#### 4.3.1 Algorithm aversion and trust in AI

One of the most salient debates in the empirical literature is whether humans display “algorithm aversion” (Dietvorst et al., 2015), that is, that they trust an algorithm's opinion less than that of a human even when the algorithm is shown to be more accurate (Kennedy et al., 2022; see also, Mahmud et al., 2022). Kennedy et al. examined the direct trust humans were willing to place in algorithms in terms of their performance and/or ability, relative to non‐algorithmic sources of advice (Kennedy et al., 2022, p. 1132). Two domains of public service were studied: forecasting geopolitical events and forecasting recidivism in criminal justice (Kennedy et al., 2022, p. 1133). While the object of trust here is the algorithm, their study also analyses a hybrid condition in which an expert has received input from an algorithm (Kennedy et al., 2022, p. 1136). This provides some information about trust‐enhancing designs of implementing AI in public institutions.

Kennedy et al. claim that their findings “refute several conclusions of the algorithm aversion literature” (Kennedy et al., 2022, p. 1144). Respondents gave greater weight to advice from algorithms than from humans, even including experts. Moreover, in the hybrid condition, an expert's decision was given greater weight if the decision was made in light of an algorithm's advice (Kennedy et al., 2022, p. 1133). However, respondents seemed to prefer at least some trained human's involvement in the decision‐making, suggesting that humans‐in‐the‐loop can strengthen trust (Kennedy et al., 2022, p. 1144). The transparency of the algorithm, however, did not have a strong impact on trust levels (Kennedy et al., 2022, p. 1144). As for criminal recidivism, algorithms created based on larger data sets and with a higher number of variables used in their models were generally preferred over those with smaller sets and fewer variables. As regards accuracy, false negatives, that is, predicting that someone will not commit a crime and they turn out to do, were seen as more problematic by respondents than false positives (Kennedy et al., 2022, p. 1144). Note that these empirical findings do not necessarily align with normative principles. Many criminal law systems will aim to avoid false positives at the cost of false negatives.

In an experiment with US residents, however, Yalcin et al. show that their perceived trust of human judges is indeed higher when compared to that of algorithmic judges (Yalcin et al., 2022). The authors measured participants' trust toward judges by aggregating four items: perceived trustworthiness, unbiasedness, fairness, and predictability (Yalcin et al., 2022, p. 7). The latter three items speak to the contextuality of trust, as being unbiased, fair, and predictable are attributes which have been associated in previous research with trust in judges and the legal system (See the references in Yalcin et al., 2022, p. 4). Yalcin et al. not only measured relative levels of perceived trust in judges, but also the intention to use the court system. This is valuable for the research interest of this article, that is, trust in institutions which are deploying AI systems. Even though participants acknowledged that algorithms might lead to quicker and cheaper processes, their study found that “perceived trust, and willingness to submit a case to court is negatively influenced by the use of algorithmic judge” (Yalcin et al., 2022, p. 16) The authors further added case complexity to their study. Complexities may arise from psychological or emotional factors or from technical issues. Interestingly, both technical and emotional complexities reduced trust in human judges, whereas only emotional but not technical complexities reduced trust in algorithmic judges (Yalcin et al., 2022, p. 16).

#### 4.3.2 Communication and initial trust in automated public services

Closely related to studies on “algorithm aversion” is research which draws on an earlier model of trust in machines (See, Lee & Moray, 1992; Lee & See, 2004). The model breaks down trust into three elements: the performance basis of trust (a machine's competency and expertise), the process basis (how the machine and the algorithms behind it operate), and the purpose basis (the intent of the machine's designer and their positive orientation toward the trustor; Aoki, 2021, p. 2; Aoki, 2020, p. 3; drawing on Lee & Moray, 1992; Lee & See, 2004). While not per se concerned with trust in institutions, some papers in the empirical batch utilize the model to arrive at insights relevant to our review (See also, Kennedy et al., 2022; see further, although not in the final sample, Spatola & MacDorman, 2021). In two related publications, Aoki studied the introduction of AI to the Japanese public sector by focusing on the public's initial trust in public services and the impact that communication around the use of AI has on that trust (Aoki, 2021, p. 7). Initial trust refers to the public's trust levels prior to interacting with an AI system, hence capturing the effects on trust at the stage of introducing the technology to the public sector (Aoki, 2021, p. 1).

The first reviewed study by Aoki concerns the introduction of and citizens' initial public trust in AI chatbots (Aoki, 2021). The paper hypothesizes that initial public trust in AI chatbots depends on the area of enquiry and on the purposes communicated to the public for introducing the technology (Aoki, 2021, p. 1). As with the research by Kennedy et al., the primary object of trust is thus the AI technology. However, the study also tests the variation in the degree of initial public trust in AI chatbots relative to the public trust in human administrators (Aoki, 2021, p. 4), thus providing insights into the changes in perceived trust once the technology is being introduced to an institution. Aoki finds that the public's trust in AI chatbots is lower for some areas of enquiry than for others, especially so in parental support: “to be trustworthy, responses in this area must provide enquirers with the information they want, employ situational judgment, and communicate with them in a socially proper and empathetic manner” (Aoki, 2021, p. 9). In other areas, namely waste separation and tax consultation the study found that “the act of responding is relatively easier to program and requires fewer social and political skills,” hence receiving higher levels of initial trust. As regards the communication of purposes, the effect sizes were small. However, if the government communicated reasons for the introduction of AI chatbots that directly benefit citizens, such as achieving uniformity in response quality and timeliness in responding, this enhanced public trust in chatbots (Aoki, 2021, p. 9).

The second reviewed study by Aoki concerns the public's initial trust in an AI decision aid utilized in the delivery of public services in the Japanese nursing care sector. The AI is supposed to create a care plan for care users (Aoki, 2021). The study focuses on two different issues of communication: first, assuring that a human is still in the decision loop and, second, stating the purposes for using the AI (Aoki, 2021, p. 7). The study finds that the proportion of those respondents who trusted a care plan prepared with AI assistance more than a care plan not involving AI was higher with the communicated assurance that a human was in the decision loop than without. Besides demonstrating the importance of communicating the involvement of humans, Aoki concludes that this also reveals the respondent's reservations about fully AI‐driven automation in care planning (Aoki, 2021, pp. 5–6). The study did not find strong support for the hypothesis that communicating the purposes for using the AI system makes a difference in respondents' reported trust levels (Aoki, 2021, pp. 5–6).

#### 4.3.3 Added value

Trust in public institutions' use of AI is also studied as regards whether the AI system provides added value to citizens. Drawing on public value creation theory (a field of public management studies), Wang et al. tested whether citizens' use of AI voice robot services can help increase citizens' perception of government transparency and trust in government, as the robot can provide “timely information and services without prejudice” (Wang et al., 2021). Their survey of Chinese citizens who had just used China's first tax AI voice robot named “Tax Baby” (Wang et al., 2021, p. 8), however, did not find a significant relationship between the use of this AI and trust in government.

Drew reports on research results which were obtained by the UK's Government Data Science Partnership—a collaboration by several UK public institutions—during their drafting process of an ethical framework for data science in government (Drew, 2016). In their research, participants' attitudes were context‐driven, and they considered the acceptability of data science in government on a case‐by‐case basis (Drew, 2016, p. 8). “Firstly, they consider the overall policy aim and likely intervention as well as whether data science provides more value than more traditional methods. Only if they accept that can they then move on to a nuanced risk assessment of balancing the level of public outcome and project efficacy against privacy and unintended consequences” (Drew, 2016, p. 8).

Drew raises another relevant point: engagement may “potentially decrease trust if too many hypothetical risks are highlighted” (Drew, 2016, p. 9). High levels of technological literacy were found to not necessarily lead to support for data science methods but at times to “suspicion about their use” (Drew, 2016, p. 9). The study suggests that engagement may be better used to understand how people value the overall policy area and the utilization of their data to solve issues within these areas rather than to build “trust in the data science method per se” (Drew, 2016, p. 9).

In earlier empirical trust research, the so‐called “Technology Acceptance Model” (TAM) developed in the field of information systems and management enjoyed popularity (Bagozzi et al., 1992). For example, Wang and Lu built a theoretical model based on TAM, hypothesizing that perceived usefulness and perceived ease of use—two factors which according to TAM foster trust in technology—will strengthen trust in e‐government (Wang & Lu, 2010). In Wang's and Lu's conceptualization, two forms of institutional trust—stated as “trust in internet” and “trust in government”—are the independent variables for trust in e‐government as well as for the perceived usefulness and perceived ease of use which are also independent variables for trust in e‐government (Wang & Lu, 2010). The term “e‐government” was frequently deployed when scholars began to address the increasing application of information and communication technology to automate public services by drawing on TAM (Wang & Lu, 2010, p. 1; see also Fu & Lee, 2014; Cho et al., 2019). This line of research suggests that AI systems which are perceived as useful and easy to use represent an added value and have a higher chance of being trusted. As regards the AI Act, this raises the question of who is going to be the primary user of AI in public institutions (see Section 2.2). Technology which public servants perceive as useful and user‐friendly may not be perceives as such by citizens.

#### 4.3.4 Political economy

Lastly, the political economy of AI was also considered in the reviewed empirical literature. In a survey on US‐American residents, Zhang and Dafoe find that Americans have only low to moderate levels of trust in governmental, corporate, and multistakeholder institutions to develop and manage AI in the public's interest (Zhang & Dafoe, 2020). Across institutions, university researchers were most trusted, followed by the US military, scientific organizations, the Partnership on AI, tech companies (excluding Facebook), and intelligence organizations; followed by US federal or state governments, and the UN; followed by Facebook (Zhang & Dafoe, 2020). One should add, however, that their survey was conducted shortly after the Facebook/Cambridge Analytica scandal was widely reported in the media in 2018 (Zhang & Dafoe, 2020, p. 191). This may likely have lowered public trust in tech companies, especially Facebook. Note, however, that in Kennedy et al.'s study the provider of the algorithm also mattered: a prestigious university was trusted more than a private company (Kennedy et al., 2022, p. 1144). Finally, in Zhang's and Dafoe's study, the individual‐level trust in various actors to responsibly develop and manage AI did not predict individual's general support for developing AI (Zhang & Dafoe, 2020, p. 188). The latter may thus be higher, despite lower trust in institutions.

#### 4.3.5 Interim findings

The review of the empirical literature has provided some further answers to RQ1, specifically under what circumstances the AI Act would be able to foster citizens' trust in AI. As mentioned, however, the results in these studies are not generalizable. With this caveat in mind, the result of Yalcin et al.'s study that the intention of citizens to use courts is affected by the deployment of AI judges points towards a risk for public institutions to lose citizens' trust when using AI. Here, the use of AI in public institutions appears to have a direct negative impact on perceived trust in the public service. Conclusive evidence about the existence of a systematic human aversion against algorithms appears to be lacking. Keeping humans in the loop, however, was shown to raise trust levels. Citizens are also likely to trust AI more if it adds some value, that is, if public institutions using AI provide services which citizens perceive as having improved.

Results were further shown to be domain specific. Where emotional factors matter such as in parental support, AI in public institutions will likely be trusted less. Some of the normative desiderata of trustworthiness were found to have only small effects, namely the communication of the reasons for the use of AI (except when failures are communicated), transparency, and technological literacy. Future research should thoroughly test these issues. For example, the finding of Kennedy et al. that false negatives in crime prediction were seen as more problematic than false positives conflicts with the normative decision in many criminal law systems to prevent wrongful convictions at the cost of preventing wrongful acquittals (see, e.g., Volokh, 1997). Trust perceptions are likely to change and further differentiate as AI continues to be used more widely. Tracking these changes empirically presupposes of course transparency about the utilization of AI in public institutions.

Research outside of the final sample seems to partly reach different conclusions than the reviewed normative literature. Drobotowicz et al. held a design workshop with citizens on trustworthy AI services in the public sector (Drobotowicz et al., 2021). They found that citizens demanded transparency and explainability of the AI processes and also valued human involvement in the decision‐making as well as control over their data (Drobotowicz et al., 2021, pp. 110–112). Consent—a pillar of trustworthiness in the normative batch of the sample—has not been studied at all in the empirical batch. Lastly, the political economy of AI seems to matter as well empirically, with participants trusting universities more than private companies as developers of AI.

As mentioned, the emergence of trust is regularly the result of correlated effects from institutional and interpersonal trust as well as trust in technology itself (cf. Section 3.3). Perceptions of the trustworthiness of AI in the public sector will often depend on institutional trust in government (Chen et al., 2021; Chen & Wen, 2021). In other words, institutional trust will serve as an independent variable for trust in AI systems. While empirical findings of what creates trust cannot replace the normative assessment of what is well‐placed trust, the findings presented in this part of the review suggest that the AI Act by itself may likely not be able to engineer trust in AI in public services. The final section concludes with comments on the normative and empirical findings in the reviewed literature with a view on the EU's regulatory approach to AI.

## 5 Open challenges for the AI act and trustworthy AI

In light of the literature review and the shown conceptual complexity and heterogeneity of trust and trustworthiness, the AI Act's goal of bringing about trustworthy AI appears overly ambitious and by itself improbable in practice. Below we state four acute challenges facing the European Commission's attempt to signal the trustworthiness of AI through its proposed regulation.

### 5.1 Uncertain antecedents of trust

Much remains unknown about the antecedents of perceived trust in public institutions which utilize AI. As mentioned, not a single study in the reviewed empirical sample has been conducted with EU citizen participants. Of course, the choice of variables for our review was rather strict. However, as an effort of trust engineering in public‐sector AI, the AI Act is tainted with uncertainty as regards its prospects of success.

The accountability mechanisms suggested in the reviewed literature appear to be empirically understudied. The effect of informed consent, for example, has so far not been addressed at all in the relevant empirical sample. The effects of transparency, to draw on another example, have so far been ambiguous. At the same time, the normative recommendation to keep humans in the loop of automated decision‐making systems finds ample support in the empirical literature. Moreover, trust proves to be context‐dependent.

The proposed AI Act aims to harmonize regulation for all AI systems by establishing a layered regulatory structure for different levels of risk. Obviously, legislation with such a wide scope needs to remain highly general. It will hardly be sensitive enough to the differential degrees to which citizens would want particular public services to be automated or not. Domains such as parental support and waste collection would currently not qualify as high‐risk areas according to Annex III of the AI Act. And yet, research on (Japanese) citizens shows different effects of using an AI to provide public services in these domains. Horizontal regulation such as the AI Act can thus only ever be a first step to signaling the trustworthiness of a particular AI system.

At the same time, many empirical surveys on people's trust in AI will be too generic to provide conclusive evidence on citizens' trust in a particular AI system deployed in the public sector (O'Neill, 2012). Survey items such as “I trust the AI science community to do what is right” (Chen & Wen, 2021, p. 122) are not differentiating between which members of the AI science community the respondent trusts and to which degree. The policy language of creating “trustworthy AI” through regulation thus tends to overstate its claim.

### 5.2 Threat of misalignment between trustworthiness and degrees of trust

As mentioned, trustworthiness and actual trust levels can be misaligned. Whenever trust in a public institution is lowered after the implementation of a “trustworthy” AI system, citizens will be disincentivized to rely on the public institution's services. Recall the lowered willingness of study participants to use courts with AI judges. Lower trust in a public institution can reduce citizens' support of the institution and their compliance, which in turn affects its ability to perform well (Nye Jr., 1997). Part of the relevant empirical research proceeds under the reasonable assumption that AI needs to add some value to citizens to be accepted. Worsening performance can diminish the perceived value of a public service and can further hurt the institution's legitimacy. However, if citizens begin to trust AI, then discriminatory and normatively “untrustworthy” AI practices may be perceived to be more justifiable compared to when humans engage in discriminatory practices (Acemoglu, 2021, p. 44). Recent empirical research suggests that discrimination by algorithm dampens outrage compared to discrimination by humans (Bigman et al., 2022). Labeling AI as “trustworthy” by law thus blurs the line between a normative benchmark of trustworthiness and actual levels of trust in AI amongst citizens.

### 5.3 Concealed behavioral factors behind the acceptability of risks

The AI Act's expertocratic model of evaluating trustworthiness through the acceptability‐of‐risk standard may further conceal the behavioral factors of trust. Citizens' trust in AI will not emerge out of fully rational deliberations, as the research on algorithm aversion illustrates. Actual human trust in AI could even prove to be gameable. The assurance that a human being is in the decision loop may increase citizens' trust even if this human has little to no effect on the decisional outcome.

Moreover, if lawmakers or AI developers decide which risks are acceptable, as suggested in the AI Act proposal, then these decisions must be trustworthy, too. By invoking the normative concept of trustworthiness, the AI Act cannot discharge the benchmark it establishes. If AI developers were to, for example, install eventually ineffective human oversight, then this move may in the short run raise levels of human trust. This outcome would however be due to psychological factors and not a sound normative assessment of the trustworthiness of AI or its regulation.

### 5.4 The need for impartial intermediaries

Lastly, well‐placed trust in AI requires citizens to be able to hold AI‐based decision‐making accountable. That someone actually exercises these accountability options is essential, too. Potentially being able to hold AI developers and users accountable does not justify trust. Trustworthiness is instead established through an iterative process. Laypeople citizens who cannot assess the evidence for trustworthiness entirely on their own must instead trust the accountability mechanisms themselves (O'Neill, 2012). This is a crucial point for AI in the public sector which is, as mentioned, both the regulator and the addressee of regulation. Transparency, for example, thus requires more than the mere provision of information. Its output must be absorbable by laypeople citizens, for example in the way in which statistical information is conveyed (O'Neill, 2012). Research has shown that counterfactual explanations can help citizens understand and challenge automated decisions without having to understand the data science behind them (Wachter et al., 2018).

Often, intermediary institutions will have to provide the accountability work. As mentioned above (see Section 4.2.7), laypeople could judge the reliability and/or trustworthiness of an AI system by the demonstrated willingness of its developer to subject the system to review by third‐party institutions. The AI Act aims to involve such institutions under the label of “notified bodies” (Art. 33 AI Act). These “independent third parties” (AI Act, p. 14). would be tasked to “verify the conformity” of high‐risk AI systems with the AI Act [Art. 33(1) AI Act]. The AI Act has thus been interpreted as proposing the establishment of a European “ecosystem for conducting AI auditing” (Mökander et al., 2022, p. 243). To be successful, however, AI auditors such as the notified bodies must be trustworthy, too.

AI auditors can play an important role, but without proper regulation they can be “captured” by industry interests [see further, Laux et al., 2021. For a similar point, see, Selbst (2021)]. One important requirement for trustworthy intermediaries will thus be their impartiality and independence. According to the proposal, notified bodies shall be “independent” from the AI provider under assessment [Art. 33(4) AI Act] and their organization and operation shall “safeguard the independence, objectivity and impartiality of their activities” [Art. 33(5) AI Act]. Prescribing independence and impartiality by law may not be enough in practice. The notified bodies would provide their assessment work by charging a “fee” (AI Act, Recital 73). They thus have an obvious economic incentive to receive commissions from AI developers. At the same time, Article 4(1) Annex VII AI Act seems to suggest that AI developers are free to select a notified body “of their choice.” There is thus a risk that notified bodies will cater to AI providers interests to receive repeat commissions (Laux et al., 2021, pp. 3–4). The severity of this risk would not least depend on the evolution of the European AI industry. If large AI developers emerge, their market power on the demand side for AI audits could aggravate capture risk (Laux et al., 2021, pp. 8–9). In this regard, it is encouraging to see that both the reviewed normative and the reviewed empirical literature address the political economy of data.

## 6 Conclusion

This article has critically examined the conceptualization of trust in the AI Act as proposed by the European Commission. It criticized the Commission's simplistic conflation of trustworthiness with the acceptability of risks. Trust research is a heterogeneous landscape, largely without a commonly shared definition of trust or a coherent research approach. Even theoretically robust, evidence‐based policymaking around “trustworthy AI” must therefore proceed on shaky foundations. The prescriptive set of variables for reviewing trust‐research developed in this article allowed us to bring some limited clarity to the diversity of approaches encountered in the literature. Our review identified common themes in the literature, and discussed the significance of the many, diverse ways in which trust in AI can be defined, measured, incentivized, and governed.

Overall, the normative literature appears to support a more participatory approach to public accountability than currently followed in the AI Act. At the same time, it problematizes the lack of technical knowledge of the average citizen and the ambiguous effects which measures such as transparency can have. The empirical literature is less coherent. However, it contains ample support for humans remaining in the AI‐supported decision loop. Trust, however, does not always emerge out of citizens' rational deliberation. Human trust in AI could thus be misguided or, worse, gamed. There remains a threat of misalignment between levels of actual trust and the trustworthiness of applied AI. The empirical literature further illustrates how domain‐specific trust in public sector AI is. This casts some doubt on the effectiveness of a horizontal regulatory law such as the AI Act. Even if passed into law, the AI Act proposal thus appears incomplete without further sectoral regulation or standardization. Additional norms would, however, face the same problems of alignment with the current state of research on trust in AI used in the public sector. Too much still remains unknown about the antecedents of people's trust in AI. Moreover, the results of individual research studies are hardly generalizable. Not a single study in the reviewed literature has been conducted with participants from the EU. More research into “trustworthy AI” is thus needed.

Finally, the AI Act's conceptual conflation conceals the need for trustworthy institutions to successfully engineer citizens' trust in AI. Take the knowledge asymmetries between AI experts and layperson citizens as an example. Average citizens' lack of understanding how AI systems work may motivate risk regulation which relies heavily on expert judgments, although the broader literature on risk regulation has been debating the paternalistic flavor of this approach for decades (see Section 2.3). Yet these factual obstacles to citizen assessments of trustworthiness are not enough to equate “trustworthy” AI with “acceptable” AI as judged by experts. As mentioned, the Commission's proposal leaves it to AI developers to self‐assess their AI systems or subject themselves to external assessments by “notified bodies” (see Section 5.4). However, laypeople citizens may read a developer's willingness to subject an AI to external review precisely as a sign of trustworthiness. It could thus well be that not the acceptability‐of‐risk judgments themselves, but their institutional environment will have the greater influence on citizen's trust in AI. At a minimum, establishing the impartiality and independence of intermediaries such as the notified bodies will thus be an essential normative element for the AI Act's prospects of engineering citizens' trust in AI.

## Data Availability

This article contains a litetature review. If requested, we would be happy to provide the search result list from the two research databases used, SCOPUS and Web of Science.

## Endnotes

^1^
Cf. the status of the legislative procedure: https://eur-lex.europa.eu/legal-content/EN/HIS/?uri=celex:52021PC0206 (last accessed December 1 2022).

^2^
On errors and biases in automated decision‐making systems used by governments, see also Green (2022, p. 1).

^3^
Cf. https://digital-strategy.ec.europa.eu/en/policies/expert-group-ai (last accessed on December 1, 2022).

^4^
Note that many but not all researchers define “distrust” as the opposite of “trust” which would mean that they are separate constructs that can co‐exist in the same person at the same time, cf. McKnight and Chervany (2001, pp. 41–43).

^5^
Our academic literature review necessarily excludes policy papers which address matters of trust in AI from its scope. See, for example, Institute of Electrical and Electronics Engineers (IEEE) (2017).

^6^
These four steps are an adaptation of the four stages of the Preferred Reporting Items for Systematic Reviews and Meta‐Analyses (PRISMA), cf. Moher et al. (2009). The PRISMA method has been previously applied in a literature review on artificial intelligence in the public sector, cf. de Sousa et al., 2019.

^7^
On Web of Science, the search field “topic” was selected, which searches title, abstract, author keywords and keywords plus. The latter are “words or phrases that frequently appear in the titles of an article's references, but do not appear in the title of the article itself”, cf. https://support.clarivate.com/ScientificandAcademicResearch/s/article/KeyWords-Plus-generation-creation-and-changes?language=en_US (last accessed on December 1, 2022).

^8^
The inclusion and exclusion criteria were adopted and slighted altered from de Sousa et al., 2019, p. 3. As “AI terms” the authors list: “intelligent factory; robots; robotic; analytic hierarchy process (AHP); artificial agents; natural language processing (NLP); multi‐agent systems (MAS); cellular automata (CA); cognitive mapping (CM); genetic algorithms (GA); artificial neural networks (ANN); machine learning (ML); rule‐based systems (RBS); case‐based reasoning (CBR); fuzzy logic; intelligent systems; expert system; sentimental analysis; clustering algorithm system (CAS); cognitive information technology; cognitive mapping; cognitive systems; digital neural networks; algorithm”, cf. de Sousa et al., 2019.

^9^
For a more recent suggestion on how to implement institutional oversight of AI in public institutions, see Green (2022).

^10^
Cf. the list of measures suggested in Wilson & van der Velden (2022, p. 7).
